# Development and internal validation of a prediction model of prostate cancer on initial transperineal template-guided prostate biopsy

**DOI:** 10.1186/s12894-021-00840-5

**Published:** 2021-04-23

**Authors:** Yuliang Chen, Zhien Zhou, Yi Zhou, Xingcheng Wu, Yu Xiao, Zhigang Ji, Hanzhong Li, Weigang Yan

**Affiliations:** 1 The Department of Urology, Peking Union Medical College Hospital, Chinese Academy of Medical Sciences, No. 1 Shuaifuyuan, Dongcheng District, Beijing, 100730 China; 2 The Department of Pathology, Peking Union Medical College Hospital, Chinese Academy of Medical Sciences, No. 1 Shuaifuyuan, Dongcheng District, Beijing, 100730 China

**Keywords:** Transperineal template-guided prostate biopsy, High-grade prostate cancer, Prediction model, Nomogram

## Abstract

**Background:**

Due to the invasiveness of prostate biopsy, a prediction model of the individual risk of a positive biopsy result could be helpful to guide clinical decision-making. Most existing models are based on transrectal ultrasonography (TRUS)-guided biopsy. On the other hand, transperineal template-guided prostate biopsy (TTPB) has been reported to be more accurate in evaluating prostate cancer. The objective of this study is to develop a prediction model of the detection of high-grade prostate cancer (HGPC) on initial TTPB.

**Result:**

A total of 1352 out of 3794 (35.6%) patients were diagnosed with prostate cancer, 848 of whom had tumour with Grade Group 2–5. Age, PSA, PV, DRE and f/t PSA are independent predictors of HGPC with p < 0.001. The model showed good discrimination ability (c-index 0.886) and calibration during internal validation and good clinical performance was observed through decision curve analysis. The external validation of CPCC-RC, an existing model, demonstrated that models based on TRUS-guided biopsy may underestimate the risk of HGPC in patients who underwent TTPB.

**Conclusion:**

We established a prediction model which showed good discrimination ability and calibration in predicting the detection of HGPC by initial TTPB. This model can be used to aid clinical decision making for Chinese patients and other Asian populations with similar genomic backgrounds, after external validations are conducted to further confirm its clinical applicability.

## Introduction

According to GLOBOCAN data in 2018, the incidence and mortality of prostate cancer ranked second and fifth, respectively, among all cancers in men [1]. Prostate biopsy is essential for the diagnosis of prostate cancer. Due to the invasive nature of biopsy, it would be very helpful if the individual risk of a positive biopsy result can be calculated through prediction models and guide clinical decision-making.

The incidence and prevalence of prostate cancer in Asian populations are significantly lower than in individuals of Caucasian and African descent [1, 2], suggesting ethnic differences in the occurrence of prostate cancer. At present, the most widely used and well-validated prediction models for prostate biopsy are the Prostate Cancer Prevention Trial Risk Calculator (PCPT-RC) and the European Randomized Study of Screening for Prostate Cancer Risk Calculator (ERSPC-RC) [3, 4]. However, the predicted risk of these models was reported to be overestimated by 20% in Chinese patients [5], which highlights the necessity of building a prediction model for Chinese patients, as well as Asian populations with similar genomic backgrounds.

Most of the existing models for the Chinese population, for example, the Chinese Prostate Cancer Risk Calculator (CPCC-RC), are based on transrectal ultrasonography (TRUS)-guided biopsy [6]. However, the transrectal approach has a higher probability of omitting tumours in the anterior prostate than does the transperineal approach [7]. On the other hand, transperineal template-guided prostate biopsy (TTPB) is reported to be effective in detecting prostate cancer in patients with multiple negative transrectal biopsies, mainly due to improved sensitivity for anterior and apical tumours [8, 9]. Risk models based on TRUS-guided biopsy may not appropriately predict the risk of prostate cancer detected by transperineal biopsy.

The main objective of our study was to develop and internally validate a prediction model for the detection of high-grade prostate cancer (HGPC) by TTPB in biopsy-naïve Chinese patients. In addition, we conducted an external validation of CPCC-RC, an existing prediction model based on TRUS-guided biopsy, to evaluate its performance in predicting TTPB results [10].

## Patients and methods

### Study population and design

We undertook a consecutive cohort study in prostate biopsy-naïve patients in our institution (Peking Union Medical College Hospital, a tertiary hospital in Beijing, China) between December 2003 and July 2019. The included patients met at least one of the following criteria: (1) prostate-specific antigen (PSA) > 4.0 ng/mL; (2) abnormal findings on DRE; and (3) imaging results indicating the suspicion of prostate cancer. We excluded patients with PSA levels > 100.0 ng/mL or any history of prostate cancer, previous biopsy, endocrine treatment, or perineal surgery. A retrospective analysis of prospectively collected clinical data was performed.

The collection and use of all participant data and biological specimens in this study was ethically approved by the Ethics Committee of Peking Union Medical College Hospital, Chinese Academy of Medical Science. All the patients included in our study signed a written informed consent.

### Procedures

As described in our previous study [11], TTPB was conducted in operation room with patients in lithotomy position. Local anesthesia was given to 3643 patients (96.0%), with 10 ml of 1% lidocaine injected intracutaneously and subcutaneously into the perineum, and another 10 mL onto the capsule to the right and left sides of the prostatic apex. The other 151 patients (4.0%) received general anesthesia due to intolerance of pain or personal choice.

A biplanar TRUS probe (SONOLINE Adara SLC Ultrasound; Siemens, Erlangen, Germany) attached to a brachytherapy stepping unit (Computerized Medical System, St. Louis, MO, USA), and a standard 0.5-cm brachytherapy template over the perineum were used to guide the transperineal biopsy. The length, width and height of the prostate were measured by ultrasound, and the prostate volume (PV) was calculated. One to four cores were taken by Bard biopsy gun (C.R. Bard, Covington, GA, USA) from each of the 11 regions [11], with more cores for larger prostates. The pathological assessment of biopsy specimens was conducted by two pathologists in our institution, one of whom has more than 10 years of experience in urological pathology.

### Pathological assessment

All the biopsy cores were evaluated by at least 2 experienced urological pathologists in our institution according to the 2014 International Society of Urological Pathology (ISUP) modified Gleason system [12]. Overall GS was assigned after a comprehensive assessment of proportion of different Gleason patterns and distribution of each core. Discordant results from the 2 pathologists were resolved through discussion. Pathology slides and reports of cancerous specimens included in our study before 2015 were reviewed and updated by pathologists.

### Outcomes and predictors

The outcome variable was the detection of HGPC, which in our study, was defined as prostate cancer with ISUP Grade Group > 1 (Gleason score > 6) because Grade Group 1 cancer is usually indolent and does not require aggressive treatments [13]. Referring to EAU guidelines [14], the two keynote risk calculators based on the Caucasian population [3, 4] and other prediction models of prostate biopsy [10, 15–17], we selected age, PSA level, PV, DRE, and free-to-total (f/t) PSA as potential predictors.

### Statistical analysis

Statistical analysis was performed with R software (http://www.r-project.org/, version 4.0, Vienna, Austria). The mean ± SD was used to describe data in a normal distribution, while the median and interquartile range were used for data in a skewed distribution. A multivariate logistic regression model was established with the detection of HGPC as the dependent variable. Age, PSA, PV, DRE, and f/t PSA were included as potential predictors. Similar to other prediction models [4, 10], natural logarithm transformation was performed for PSA and PV to achieve better linearity with logit P. Independent variables that have a significant impact on the detection rate in univariate analysis were included in the final models after consideration of their clinical utility. A nomogram was developed based on significant predictors. Internal validation was performed with the concordance index (c-index) calculated and calibration curve depicted for the prediction model by bootstrapping with 1000 resamples. Decision curve analysis was conducted to evaluate the clinical performance of our models. A two-sided P value < 0.05 denoted statistical significance.

## Results

### Characteristics of participants

A total of 3794 patients were enrolled consecutively from December 2003 to July 2019. The characteristics of all patients are shown in Table 1. An average of 22.2 cores were taken from each patient with a median of 22 cores. 1352 out of 3794 (35.6%) patients were diagnosed with prostate cancer, 848 of whom had tumours categorized as Grade group 2–5 according to ISUP consensus.

**Table 1:** Characteristics of 3794 men in the development cohort

Parameter	Development cohort (n = 3794)
Age (years)*	68 (61–74)
PSA (ng/ml)*	10.00 (6.80–15.78)
Abnormal DRE	831 (21.9%)
f/t PSA*	0.143 (0.098–0.200)
Prostate volume (ml)*	45 (35–60)
PC detected	1352 (35.6%)
ISUP grade group	
1	504
2	279
3	206
4	151
5	212
HGPC detected	848 (22.4%)
In PC patients	587 (43.4%)
In HGPC patients	456 (53.8%)

*Data in skewed distribution described by median and interquartile range

### Model development

**Fig. 1:**
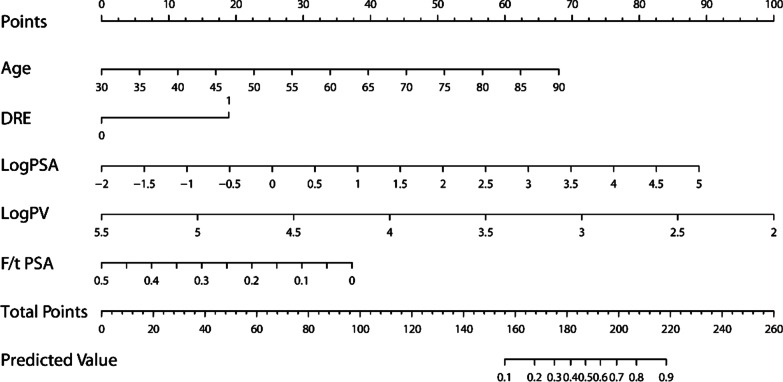
Nomogram for predicting the detection of high-grade prostate cancer by initial transperineal template-guided prostate biopsy

Age, logPSA, logPV, age, f/t PSA and DRE were significantly related to the detection of HGPC in univariate analysis and were also independent predictors of HGPC in multivariate logistic analysis (Table 2). To facilitate clinical use, we established a nomogram based on this prediction model (Fig. 1). The optimal cut-off of risk threshold which maximized Youden index (sensitivity + specificity − 1) was calculated to be 0.244 (sensitivity = 0.839, specificity = 0.748).

**Table 2:** Model predicting the detection of high-grade prostate cancer on initial transperineal template-guided prostate biopsy

Predictor	Univariate analysis			Multivariable model		
	OR(95% CI)	β	P	Adjusted OR (95% CI)	β	P
Age	1.0691 (1.0589–1.0794)	0.0668	< 0.001	1.0686 (1.0563–1.0809)	0.0663	< 0.001
DRE	7.9510 (6.6878–9.4529)	2.0733	< 0.001	3.9141 (3.1915–4.8002)	1.3646	< 0.001
logPSA	3.7242 (3.3035–4.1985)	1.3149	< 0.001	2.8486 (2.4842–3.2665)	1.0468	< 0.001
logPV	0.2128 (0.1731–0.2618)	− 1.5470	< 0.001	0.1728 (0.1323–0.2257)	− 1.7556	< 0.001
f/t PSA	0.0006 (0.0002–0.0023)	− 7.2973	< 0.001	0.0305 (0.0073–0.1276)	− 3.4910	< 0.001
Intercept	–	–	–	–	− 1.7803	–

### Internal validation

**Fig. 2:**
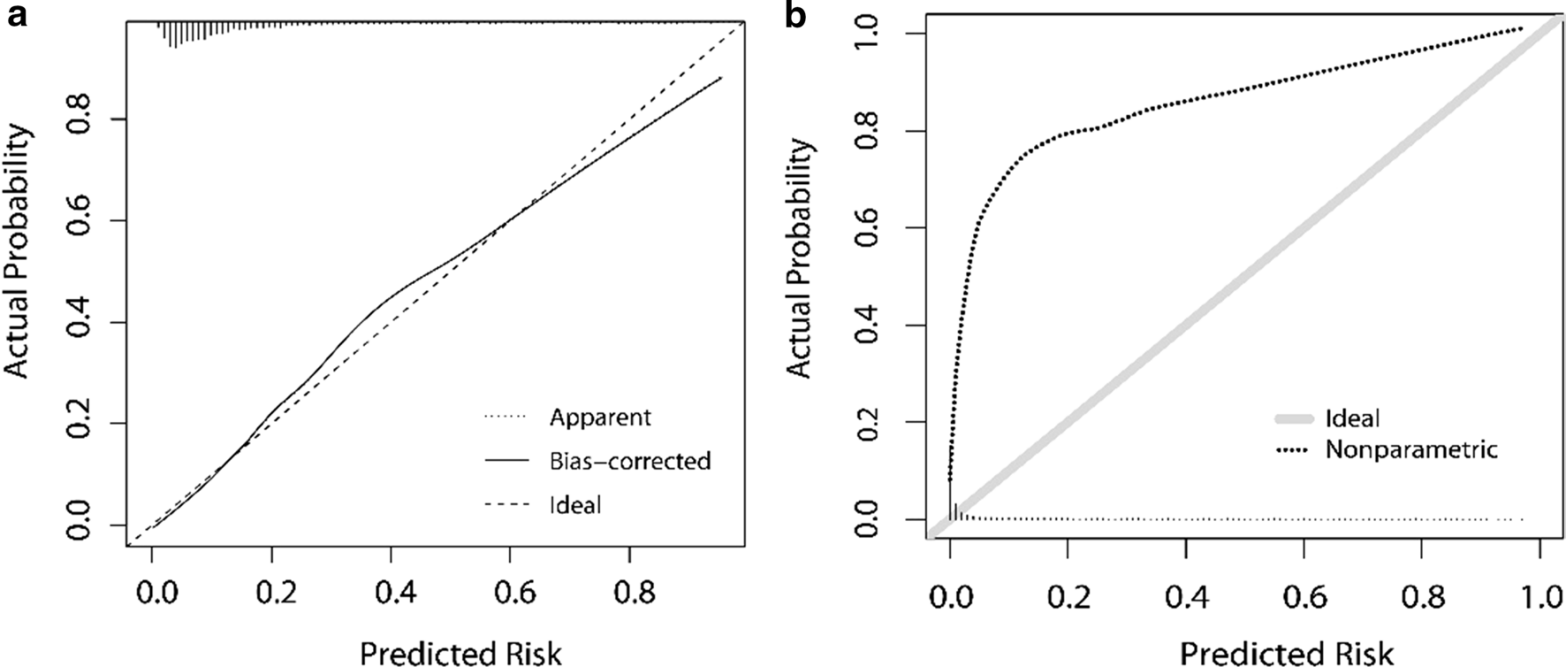
Calibration curves for the prediction models. **a** Our model in internal validation by 1000-resample bootstrapping. **b** The CPCC-RC in external validation using TTPB data

The apparent C-index of our model is 0.866. Using 1000-resample bootstrapping, the optimism of our model is calculated to be 0.002, and the bias-corrected C-index to be 0.865, demonstrating good discrimination ability. The calibration curve, also depicted by bootstrapping, is shown in Fig. 2a with excellent calibration between predictions and observations (bias-corrected slope = 0.994, bias-corrected intercept =  −0.004).

### External validation of an existing model

Using our TTPB data, we conducted external validation of CPCC-RC [10], which was an existing prediction model for the Chinese population based on a transrectal approach. The C-index was 0.826 when predicting HGPC using the CPCC-RC. The upwardly curved calibration curve in Fig. 2b suggests that the CPCC-RC underestimates the risk of HGPC in patients who undergo TTPB.

### Decision curve analysis

**Fig. 3:**
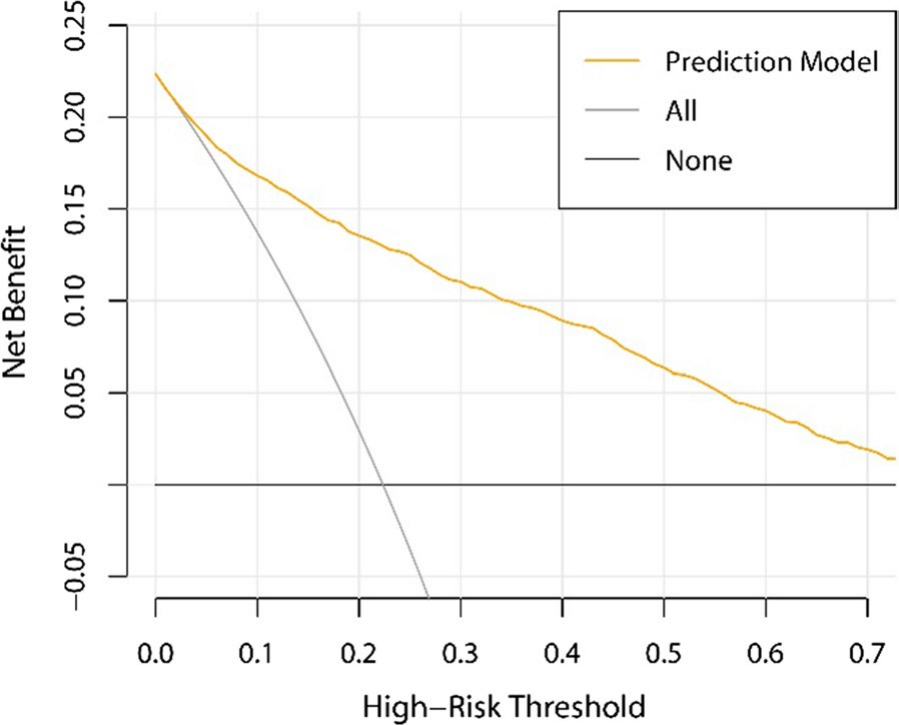
Decision curve analysis

We performed decision curve analysis for our model. In Fig. 3, the net benefit of conducting biopsy for all or none of the patients, which are two extreme situations, is represented by the grey line and the horizonal black line, respectively. In a wide range of risk thresholds, our model outperformed the two extreme strategies with a much higher net benefit. For example, if we use 0.3 as a risk threshold to determine whether TTPB is required according to our model, after weighing the benefit and cost, there is a net benefit for 11 out of every 100 people.

## Discussion

Although there have been prediction models for the detection of HGPC in Chinese population [10, 16–18], our study is the first to be based on TTPB to the best of our knowledge. Compared with the transrectal approach, transperineal biopsy is less likely to cause rectal bleeding (RR = 0.02, 95% CI 0.01–0.06) and fever (RR = 0.26, 95% CI 0.14–0.28) according to a meta-analysis by Xiang et al. [7]. A recent research based on UK National Health Service data demonstrated that from 2017 to 2019, the sepsis rate of transperineal biopsy was significantly lower than that of transrectal approach (0.42% vs. 1.12%, p < 0.001) [19]. On the other hand, transperineal biopsy was reported to be associated with severer pain (VAS score: 4.0 vs. 2.0), higher rate of additional anesthesia (15.0% vs. 1.2%) and extended operation time (17.51 ± 3.33 min vs. 14.73 ± 3.25 min) in comparison with transrectal approach [20].

While no significant difference was observed between the positive rate of transrectal and transperineal biopsy according to a meta-analysis [7], there has been evidence that the transperineal approach exceeds transrectal biopsy in terms of accurate diagnosis and risk assessment of prostate cancer [21–23]. A multi-centre autopsy study revealed that the proportion of anterior tumours did not significantly differ from that of posterior ones [24]. A higher detection rate of TTPB than of TRUS-guided biopsy was observed for anterior prostate cancer, possibly due to the difference in sites where the biopsy cores are taken [23]. When TTPB was given to active surveillance patients within 12 months of diagnosis by TRUS biopsy, histopathological upgrading was observed in 38.8% (83/208) of them by Voss et al. [21]. TTPB also demonstrated better concordance with radical prostatectomy pathology than did TRUS biopsy [22].

Although these two approaches were not directly compared in our study, we conducted external validation of the CPCC-RC. With our data collected from a similar population, the predicted risk of HGPC is underestimated by the CPCC-RC for a wide range of risk thresholds, suggesting that TTPB might be more sensitive in detecting HGPC. Of course, we must take into account the presence of different operators and pathologists between our cohort and the CPCC-RC, which may have influenced biopsy results.

MRI-targeted biopsy was reported to surpasses systematic biopsy in detecting high-grade prostate cancer [25] but still omits approximately 10% of clinically significant prostate cancer in patients with MRI-visible lesions [26]. As a result, systematic biopsies are typically suggested in addition to the MRI targeted biopsies. Meanwhile, due to the requirement of equipment and the high cost of MRI, patients with poor medical resource or poor financial conditions may not have access to MRI examination at the first visit or simply not be willing to receive one. For them, available clinical data are age, PSA level, prostate volume, and DRE result. Our models can be used during such biopsy counselling, when benefit and harm can be weighed by doctors and patients through the predicted probability of a positive biopsy result. With data from the developing cohort, we estimated the risk threshold for recommending TTPB to be 24.4%, which is in concordance with the empirical threshold of 25% [4]. This threshold can also be personalized during consultation. For patients with low predicted risk, observation might be a choice. Average risk patients might benefit from MRI for further risk assessment and high-risk patients might be recommended for MRI and biopsies.

Our study does have some limitations. First, some significant small tumours may have been missed in TTPB. In our study, this rate is not known, since it is not feasible to compare TTPB result with post prostatectomy or transperineal saturation biopsy results for each patient. Hence, some patients might need some follow up or further investigations even if the predicted risk is low. Second, some novel clinical indicators with better effects, such as Prostate Health Index, 4-Kallikrein Panel Score and Genomic Score [27], were not included in our study. Considering that such indicators are difficult to obtain in areas with general medical conditions, those included in our study are closer to practical applications. Finally, participants of our research were enrolled from a single centre, and further external validation is required to confirm the clinical applicability of our model.

## Conclusion

We established and internally validated a prediction model for the detection of HGPC on initial TTPB in a Chinese population. Good clinical performance was indicated by decision curve analysis. External validations are required to further confirm the efficacy of our model.

## Data Availability

The datasets used and analysed during the current study are available from the corresponding author on reasonable request.
